# Autonomic Nervous System Phenotyping Across Chronic Demyelinating Peripheral Neuropathies: A Comparative Study

**DOI:** 10.1111/jns.70135

**Published:** 2026-06-19

**Authors:** Bogdan Bjelica, Teodora Todorovic, Ivo Bozovic, Aleksa Palibrk, Vladimir Jankovic, Ivana Basta, Stojan Peric

**Affiliations:** ^1^ Department of Neurology Hannover Medical School Hannover Germany; ^2^ PRACTIS Clinician Scientist Program, Dean's Office for Academic Career Development Hannover Medical School Hannover Germany; ^3^ Neurology Clinic, University Clinical Center of Serbia Belgrade Serbia; ^4^ Neurology Clinic, University Clinical Center Kragujevac Kragujevac Serbia; ^5^ Faculty of Medicine, University of Belgrade Belgrade Serbia

**Keywords:** autonomic dysfunction, Charcot–Marie–Tooth disease Type 1A, chronic demyelinating polyneuropathy, chronic inflammatory demyelinating polyneuropathy, hereditary neuropathy with liability to pressure palsies, monoclonal gammopathy of undetermined significance‐associated neuropathy

## Abstract

**Background and Aims:**

This study aimed to systematically phenotype autonomic nervous system (ANS) involvement in patients with chronic inflammatory demyelinating polyneuropathy (CIDP), monoclonal gammopathy of undetermined significance‐associated neuropathy (MGUS‐PNP), Charcot–Marie–Tooth disease Type 1A (CMT1A), and hereditary neuropathy with liability to pressure palsies (HNPP).

**Methods:**

Autonomic symptoms were assessed using the SCales for Outcomes in Parkinson's Disease‐Autonomic Dysfunction (SCOPA‐AUT). Muscle strength and functional disability were evaluated using the Medical Research Council (MRC) scale, the Inflammatory Neuropathy Cause and Treatment (INCAT) disability scale, and the Overall Neuropathy Limitation Scale (ONLS).

**Results:**

A total of 343 participants were included: 98 with CIDP (mean age: 59.2 ± 13.2 years), 51 with MGUS‐PNP (66.0 ± 11.3 years), 51 with CMT1A (51.2 ± 13.1 years), 18 with HNPP (40.6 ± 15.1 years), and 125 healthy controls (58.2 ± 13.3 years). Compared with healthy controls, patients with CIDP, MGUS‐PNP, and CMT1A showed significantly higher total SCOPA‐AUT scores (*p* < 0.01). Distinct, disease‐specific ANS symptom patterns were observed across neuropathy subtypes. Overall disability was independently associated with overall autonomic symptom burden in MGUS‐PNP (*β* = 0.42, *p* < 0.05) and CMT1A (*β* = 0.68, *p* < 0.05). In CIDP, patients with active disease showed higher autonomic symptom burden than those with inactive disease (12.7 ± 11.7 vs. 8.6 ± 7.9, *p* = 0.042).

**Interpretation:**

Patients with CIDP, MGUS‐PNP, and CMT1A exhibit a substantial autonomic symptom burden with distinct disease‐specific ANS patterns. These findings highlight the relevance of autonomic dysfunction in immune‐mediated and hereditary neuropathies and warrant further studies to clarify their clinical and prognostic significance.

## 1 Introduction

The autonomic nervous system (ANS) is responsible for most aspects of visceral regulation and can be divided into central and peripheral components [1]. Peripheral ANS ganglia are located outside the central nervous system; they receive input from the central ANS via thinly myelinated preganglionic fibers and send unmyelinated postganglionic fibers to innervate internal organs and regulate visceral function [1]. Accordingly, autonomic peripheral neuropathy may manifest with a broad spectrum of symptoms, including cardiovascular, gastrointestinal, urogenital, thermoregulatory, sudomotor, and pupillomotor dysfunction [2].

Under the umbrella of peripheral neuropathies, a distinct subgroup is defined by selective or predominant involvement of autonomic fibers. Autonomic dysfunction may be secondary to systemic diseases such as diabetes mellitus, chronic alcoholism, amyloidosis, infections, paraneoplastic syndromes, or Sjögren syndrome, or present as a primary idiopathic disorder [3, 4]. As autonomic nerve fibers are predominantly thinly myelinated or unmyelinated, autonomic involvement is well recognized in primarily axonal neuropathies, such as diabetic polyneuropathy and hereditary sensory and autonomic neuropathies [2]. In acute demyelinating polyneuropathies, including Guillain–Barré syndrome, dysautonomia is also a well‐established feature [5]. In contrast, autonomic dysfunction in chronic demyelinating polyneuropathies remains less well understood.

Chronic inflammatory demyelinating polyneuropathy (CIDP) and monoclonal gammopathy of undetermined significance (MGUS)‐associated neuropathy (MGUS‐PNP) are chronic immune‐mediated polyneuropathies that share overlapping clinical and electrophysiological features, including progressive sensorimotor deficits and demyelinating patterns on nerve conduction studies [6]. Charcot–Marie–Tooth disease Type 1A (CMT1A) and hereditary neuropathy with liability to pressure palsies (HNPP) are hereditary demyelinating polyneuropathies caused by copy number variations of the *PMP22* gene [2]. Although impairment of the ANS has also been increasingly recognized in chronic demyelinating polyneuropathies [2], no study has systematically evaluated disease‐specific ANS patterns.

Therefore, the aim of this study was to conduct a systematic ANS assessment and phenotyping in patients with CIDP, MGUS‐PNP, CMT1A, and HNPP, and to compare these with healthy controls (HC). Furthermore, we examined associations between autonomic dysfunction and sociodemographic as well as clinical characteristics within each disease group.

## 2 Materials and Methods

The study was approved by the Ethics Committee of the University Clinical Center of Serbia (no. 1322/II‐4 and 17/I‐5) and was conducted in accordance with the Declaration of Helsinki and the relevant guidelines and regulations. All participants provided written informed consent. In this cross‐sectional study, we included patients with CIDP, MGUS‐PNP, CMT1A, and HNPP who were diagnosed and treated at the Neurology Clinic, University Clinical Center of Serbia. Patients were eligible for inclusion if they were 18 years of age or older and had either a confirmed duplication or deletion of the 17p11.2 chromosomal region containing the *PMP22* gene or a confirmed diagnosis of CIDP or MGUS‐PNP according to the corresponding European Federation of Neurological Societies/Peripheral Nerve Society (EFNSN/PNS) diagnostic criteria [7, 8]. Patients receiving beta‐blockers, alpha‐1 adrenergic antagonists, antidepressants, or anticholinergic drugs were excluded due to their potential effects on autonomic function (*n* = 36). The following data were collected: sex, age at the time of testing, disease duration, presence of comorbidities, as well as CIDP variant, presence and type of paraprotein, and current treatment regimens in patients with immune‐mediated polyneuropathies. The four disease groups were subsequently compared with HC. HC were eligible for inclusion only if they had no relevant medical conditions. They were selected among employees of the Neurology Clinic, the University Clinical Center of Serbia, and their relatives.

### 2.1 Autonomic Symptom Phenotyping

Autonomic symptoms were evaluated using the Serbian version of the SCales for Outcomes in Parkinson's Disease‐Autonomic Dysfunction (SCOPA‐AUT). This validated questionnaire includes 23 items scored from zero (“never”) to three (“often”), yielding a maximum total score of 69, with higher scores reflecting more pronounced autonomic impairment. The instrument assesses six autonomic domains: gastrointestinal (seven items), urinary (six items), cardiovascular (three items), thermoregulatory (four items), pupillomotor (one item), and sexual (two items for men and two items for women) [9]. Use of the Serbian version was authorized by the Movement Disorder Society.

### 2.2 Assessment of Muscle Strength, Disease Severity/Activity, and Functional Disability

Muscle strength was measured using the Medical Research Council (MRC) scale (0–5) in all patients. The MRC sum score (MRC‐SS) was derived from bilateral assessment of shoulder abductors, elbow flexors, wrist extensors, hip flexors, knee extensors, and ankle dorsiflexors [10].

In patients with immune‐mediated polyneuropathies (CIDP and MGUS‐PNP), functional disability was evaluated using the Inflammatory Neuropathy Cause and Treatment (INCAT) disability scale. This instrument assesses limitations in daily activities by separately scoring upper and lower limb function, yielding a total score ranging from 0 (*no disability*) to 10 (*severe disability*), with higher scores indicating greater functional impairment [11]. In CIDP patients, disease activity was assessed using the Chronic Demyelinating Polyneuropathy Activity Scale (CDAS). The scale classifies patients into five categories based on clinical course and treatment status, such as “cured,” “remission”, “stable active disease,” “improving,” or “unstable active disease” [12]. Patients were further grouped into inactive CIDP (“cured” and “remission”) and active CIDP (“stable active disease,” “improving,” and “unstable active disease”).

In patients with hereditary polyneuropathies (CMT1A and HNPP), functional disability was assessed using the Overall Neuropathy Limitation Scale (ONLS) [13]. This scale evaluates limitations in upper and lower limb function as well as the ability to perform daily activities, with higher scores indicating greater functional disability. In addition, disease severity was evaluated using the Charcot–Marie–Tooth Examination Score (CMTES), a validated instrument based on clinical symptoms and neurological examination findings, where higher scores reflect more severe neuropathy [14].

### 2.3 Statistical Analysis

Statistical analyses and graphical representations were performed using IBM SPSS Statistics (version 28; IBM Corp., Chicago, IL, USA) and GraphPad Prism (version 10.6.1; GraphPad Software, San Diego, CA, USA). Data normality was assessed with the Shapiro–Wilk and Kolmogorov–Smirnov tests, along with graphical inspection of histograms. Data are presented as violin plots with all individual data points displayed. A two‐sided *p* < 0.05 was considered statistically significant, while *p* < 0.01 and *p* < 0.001 were considered highly and very highly statistically significant, respectively. Group comparisons were conducted using the *χ* ^2^ test and Kruskal–Wallis tests with Dunn's multiple comparisons. Correlations were assessed using Spearman's rank correlation coefficient.

Linear regression analyses were performed to examine factors associated with overall autonomic symptom burden (total SCOPA‐AUT as dependent variable). Analyses were conducted separately for CIDP, MGUS‐PNP, and CMT1A patients. Multivariable models included sex, age, muscle strength (MRC‐SS), and disease‐specific disability measures (total INCAT score for CIDP and MGUS; ONLS and CMTES for CMT1A). Models were estimated using the enter method.

## 3 Results

A total of 343 participants were included. The cohort comprised 98 patients with CIDP (60% male; mean age 59.2 ± 13.2 years), 51 with MGUS‐PNP (67% male; 66.0 ± 11.3 years), 51 with CMT1A (41% male; 51.2 ± 13.1 years), 18 with HNPP (50% male; 40.6 ± 15.1 years), and 125 HC (48% male; 58.2 ± 13.3 years). The main sociodemographic and clinical characteristics of the cohort are presented in Table 1 and Table S1. Among patients with immune‐mediated polyneuropathies, most were receiving oral corticosteroids or IVIG. Patients with CIDP were predominantly in remission on treatment, followed by remission off treatment, while only 10% had unstable active disease. Among patients with hereditary neuropathies, those with CMT1A were more severely affected and exhibited greater functional disability than those with HNPP.

**TABLE 1:** Main sociodemographic and clinical characteristics of included participants.

Features	CIDP	MGUS	CMT1A	HNPP	HC	*p* *
*N*	98	51	51	18	125	
Sex (% of males)	60.2%	66.7%	41.2%	50.0%	48.0%	**0.040**
Age at the time of testing (years, mean ± SD)	59.2 ± 13.2	66.0 ± 11.3	51.2 ± 13.1	40.6 ± 15.1	58.2 ± 13.3	**< 0.001**
Disease duration (years, mean ± SD)	8.2 ± 6.3	4.6 ± 3.8	21.4 ± 15.6	18.8 ± 16.2	—	**< 0.001**
Presence of comorbidities (*N*, %)						
Diabetes	17, 17.3%	10, 19.6%	3, 5.9%	0, 0.0%	—	**0.045**
Connective tissue diseases	0, 0.0%	2, 3.9%	0, 0.0%	0, 0.0%	—	—
Other autoimmune diseases	3, 3.1%	3, 5.9%	0, 0.0%	5, 27.8%	—	**< 0.001**
MRC‐SS (mean ± SD)	54.3 ± 7.3	53.2 ± 13.2	49.5 ± 6.4	54.9 ± 5.6	—	**0.002**
Immune‐mediated polyneuropathies						
CIDP variant (*N*, %)						
Typical	61, 62.2%	—	—	—	—	—
Pure sensory	9, 9.2%	—	—	—	—	—
Pure motor	9, 9.2%	—	—	—	—	—
Distal	8, 8.2%	—	—	—	—	—
Lewis‐Sumner syndrome	4, 4.1%	—	—	—	—	—
Paraparetic	7, 7.1%	—	—	—	—	—
Focal	0, 0.0%	—	—	—	—	—
CIDP EFNS/PNS diagnostic criteria (*N*, %)						
Definite	87, 88.8%	—	—	—	—	—
Probable	1, 1.0%	—	—	—	—	—
Possible	10, 10.2%	—	—	—	—	—
Paraprotein detected (*N*, %)						
No	89, 90.8%	0, 0.0%	—	—	—	—
IgG	5, 5.2%	14, 27.5%	—	—	—	—
IgA	2, 2.0%	32, 62.7%	—	—	—	—
IgM	0, 0.0%	2, 3.9%	—	—	—	—
Missing	2, 2.0%	3, 5.9%	—	—	—	—
Prior treatment (*N*, %)						
Oral corticosteroid therapy	84, 85.7%	27, 52.9%	—	—	—	**< 0.001**
Pulse corticosteroid therapy	20, 20.4%	5, 9.8%	—	—	—	0.112
IVIG	41, 41.8%	6, 11.8%	—	—	—	**< 0.001**
TPE	5, 5.1%	2, 3.9%	—	—	—	1.000
Other immunosuppressive therapy (*N*, %)						0.858
No	85, 86.7%	46, 90.2%	—	—	—	
Azathioprine	10, 10.2%	4, 7.8%	—	—	—	
Cyclophosphamide	2, 2.0%	1, 2.0%	—	—	—	
Both	1, 1.0%	0, 0.0%	—	—	—	
Current disease activity according to CDAS (*N*, %)						
Remission (off treatment)	25, 25.5%	—	—	—	—	—
Remission (on treatment)	38, 38.8%	—	—	—	—	—
Stable active disease	19, 19.4%	—	—	—	—	—
Improving	6, 6.1%	—	—	—	—	—
Unstable active disease	10, 10.2%	—	—	—	—	—
Inactive CIDP	62, 63.3%	—	—	—	—	—
Active CIDP	36, 36.7%	—	—	—	—	—
INCAT score (mean ± SD)						
Upper limbs	0.7 ± 0.9	1.2 ± 1.0	—	—	—	**< 0.001**
Lower limbs	1.2 ± 1.4	1.2 ± 1.0	—	—	—	0.398
Total	1.9 ± 2.1	2.4 ± 1.8	—	—	—	**0.021**
Hereditary polyneuropathies						
CMTES score (mean ± SD)	—	—	11.3 ± 5.5	7.8 ± 4.5	—	**0.015**
ONLS score (mean ± SD)						
Upper limbs	—	—	1.5 ± 1.2	0.9 ± 1.0	—	0.081
Lower limbs	—	—	2.0 ± 0.8	0.9 ± 0.9	—	**< 0.001**
Total	—	—	3.5 ± 1.7	1.8 ± 1.8	—	**0.002**

*Note:* Post hoc analyses are presented in Table S1.

Abbreviations: CDAS, chronic inflammatory demyelinating polyneuropathy disease activity status; CIDP, chronic inflammatory demyelinating polyneuropathy; CMT1A, Charcot–Marie–Tooth Type 1A; CMTES, Charcot–Marie–Tooth Examination Score; HC, healthy controls; HNPP, hereditary neuropathy with liability to pressure palsies; INCAT, inflammatory neuropathy cause and treatment; IVIG, intravenous immunoglobulins; MGUS, monoclonal gammopathy of undetermined significance; MRC‐SS, Medical Research Council sum score; *N*, number; ONLS, Overall Neuropathy Limitations Scale; SD, standard deviation; TPE, therapeutic plasma exchange.

* *p* values when comparing all groups; Bold values indicate statistically significant differences between groups (*p* < 0.05).

### 3.1 Autonomic Dysfunction in Chronic Demyelinating Polyneuropathies vs. HC

**FIGURE 1:**
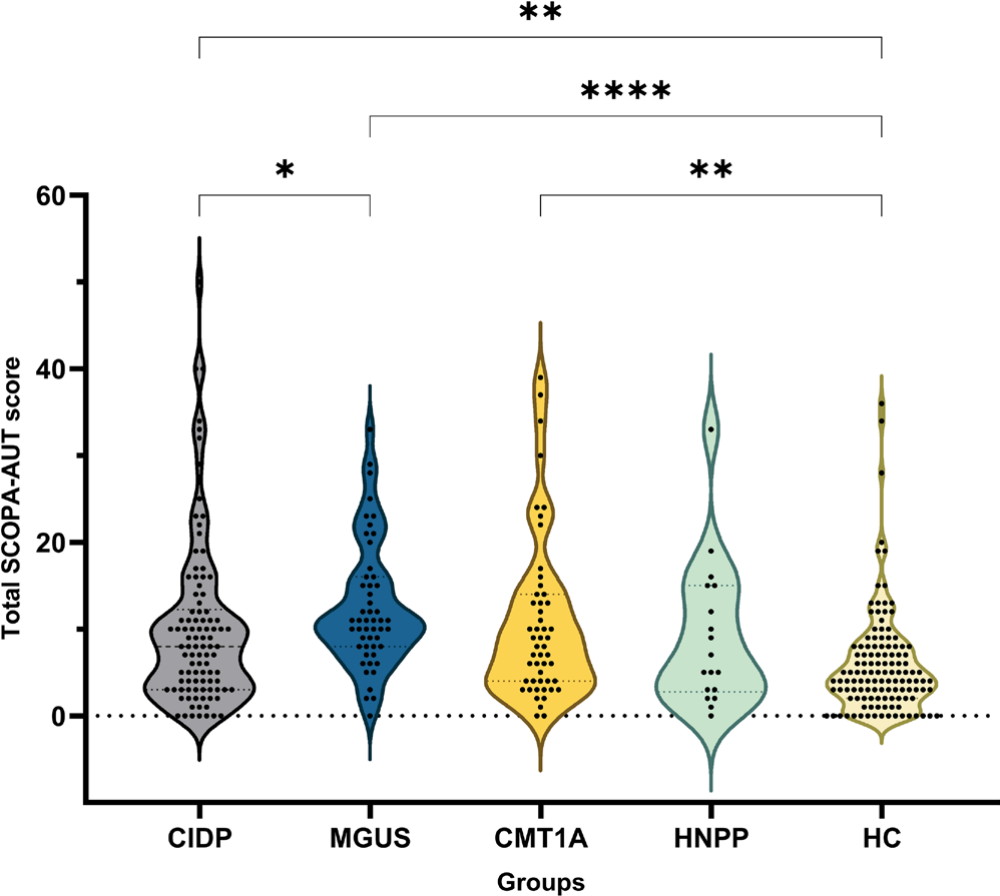
Overall autonomic symptom burden in patients with CIDP, MGUS‐PNP, CMT1A, HNPP, and HC. *p_adjusted_ < 0.05; **p_adjusted_ < 0.01; ****p_adjusted_ < 0.0001; group differences were analyzed using Kruskal–Wallis tests with Dunn's multiple comparisons. Data are presented as violin plots; dots represent individual patients. CIDP, chronic inflammatory demyelinating polyneuropathy; CMT1A, Charcot–Marie–Tooth Type 1A; HC, healthy controls; HNPP, hereditary neuropathy with liability to pressure palsies; MGUS, monoclonal gammopathy of undetermined significance‐associated polyneuropathy; SCOPA‐AUT, SCales for Outcomes in Parkinson's Disease‐Autonomic Dysfunction.

Figure 1 illustrates the overall autonomic symptom burden in patients with CIDP, MGUS‐PNP, CMT1A, HNPP, and HC. Compared with HC, patients with CIDP, MGUS‐PNP, and CMT1A had significantly higher total SCOPA‐AUT scores (CIDP vs. HC: 10.1 ± 9.6 vs. 5.9 ± 6.0, *p* = 0.001; MGUS‐PNP vs. HC: 12.5 ± 7.2 vs. 5.9 ± 6.0, *p* < 0.0001; CMT1A vs. HC: 10.7 ± 9.4 vs. 5.9 ± 6.0, *p* = 0.002), whereas no significant difference was observed for HNPP (*p* < 0.05). Furthermore, patients with MGUS‐PNP exhibited significantly higher overall autonomic symptom burden in comparison to CIDP patients (MGUS‐PNP vs. CIDP: 12.5 ± 7.2 vs. 10.1 ± 9.6, *p* = 0.036).

**FIGURE 2:**
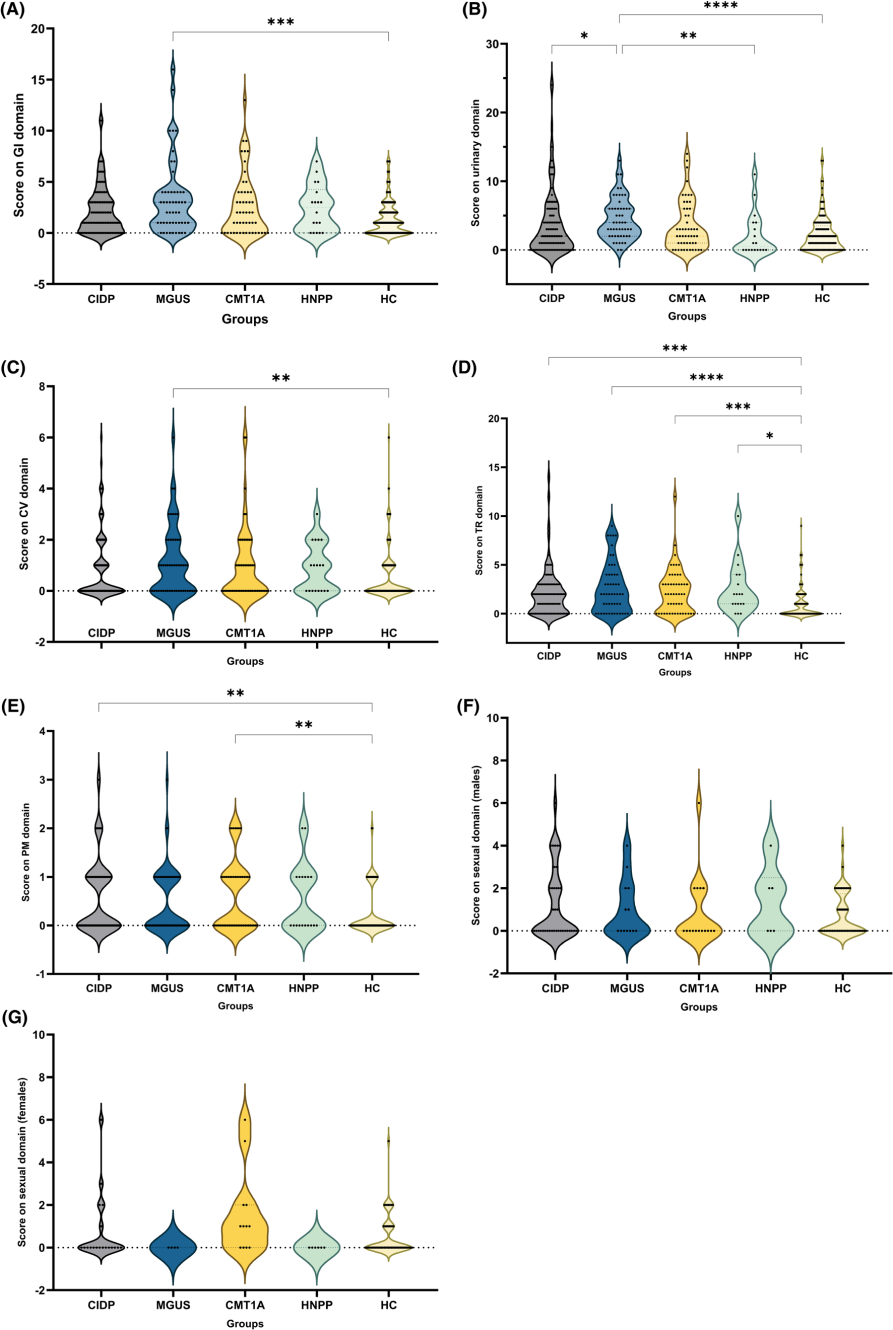
(A–G) Overall autonomic symptom burden in specific domains of autonomic function in patients with CIDP, MGUS‐PNP, CMT1A, HNPP, and HC. *p_adjusted_ < 0.05; **p_adjusted_ < 0.01; ****p_adjusted_ < 0.0001; group differences were analyzed using Kruskal–Wallis tests with Dunn's multiple comparisons; data are presented as violin plots. CIDP, chronic inflammatory demyelinating polyneuropathy; CMT1A, Charcot–Marie–Tooth Type 1A; CV, cardiovascular; GI, gastrointestinal; HC, healthy controls; HNPP, hereditary neuropathy with liability to pressure palsies; MGUS, monoclonal gammopathy of undetermined significance‐associated polyneuropathy; PM, pupillomotor; SCOPA‐AUT, SCales for Outcomes in Parkinson's Disease‐Autonomic Dysfunction; TR, thermoregulatory.

Figure 2A–G depicts the autonomic symptom burden across specific autonomic domains in patients with CIDP, MGUS‐PNP, CMT1A, HNPP, and HC. In the gastrointestinal domain, MGUS‐PNP patients scored significantly worse than HC (3.3 ± 3.6 vs. 1.4 ± 1.7, *p* = 0.0009). In the urinary domain, MGUS‐PNP patients also showed significantly higher symptom burden compared to HC (4.5 ± 3.0 vs. 2.3 ± 2.6, *p* < 0.0001). In addition, MGUS‐PNP patients had worse urinary scores compared to CIDP (4.5 ± 3.0 vs. 3.8 ± 4.9, *p* = 0.026) and HNPP patients (4.5 ± 3.0 vs. 2.3 ± 2.6, *p* = 0.004). In the cardiovascular domain, the only significant difference was observed between MGUS‐PNP patients and HC, with MGUS‐PNP patients showing higher scores (1.1 ± 1.3 vs. 0.5 ± 1.0, *p* = 0.0075). In the thermoregulatory domain, CIDP (2.0 ± 2.4 vs. 1.0 ± 1.6, *p* = 0.0001), MGUS‐PNP (2.7 ± 2.6 vs. 1.0 ± 1.6, *p* = 0.0001), and CMT1A patients (2.3 ± 2.3 vs. 1.0 ± 1.6, *p* = 0.0002) showed significantly worse scores compared with HC. In the pupillomotor domain, CIDP (0.6 ± 0.8 vs. 0.2 ± 0.5, *p* = 0.0022) and CMT1A patients (0.6 ± 0.7 vs. 0.2 ± 0.5, p = 0.0075) had higher scores than HC. Finally, no significant differences were observed in male or female sexual function between any patient group and HC.

### 3.2 Subgroup Analyses—Clinical and Sociodemographic Correlates of Autonomic Dysfunction

In CIDP, women exhibited significantly higher thermoregulatory domain scores compared with men (2.7 ± 3.0 vs. 1.6 ± 1.7, *p* = 0.033). CIDP patients with active disease had significantly higher overall autonomic symptom burden in comparison to CIDP patients with inactive disease (12.7 ± 11.7 vs. 8.6 ± 7.9, *p* = 0.042). The presence of diabetes mellitus or other autoimmune diseases was not associated with differences in any autonomic domain (all *p* > 0.05). Likewise, no differences were observed according to CIDP variant, EFNS/PNS diagnostic certainty, prior or ongoing immunomodulatory treatment, age at the time of testing, or disease duration (all *p* > 0.05). Higher autonomic symptom burden was associated with greater muscle weakness as measured by the MRC‐SS (*ρ* = −0.41, *p* < 0.001) and with increased functional disability, including higher INCAT upper limb scores (*ρ* = 0.40, *p* < 0.001), INCAT lower limb scores (*ρ* = 0.35, *p* < 0.001), and total INCAT scores (*ρ* = 0.40, *p* < 0.001). In a multivariable linear regression model adjusted for age, sex, muscle strength, and functional disability, none of the included variables (age, sex, MRC‐SS, total INCAT score) were independently associated with overall autonomic symptom burden (Table S2A). Nevertheless, the overall model was statistically significant and explained approximately 16% of the variance in overall autonomic symptom burden (adjusted *R* ^2^ = 0.155, *p* < 0.001).

In MGUS‐PNP, male patients exhibited significantly greater ANS involvement than females, with higher impairment in the urinary domain (5.3 ± 3.2 vs. 3.1 ± 1.9, *p* = 0.017), cardiovascular domain (1.4 ± 1.5 vs. 0.5 ± 0.7, *p* = 0.036), and overall autonomic dysfunction (14.0 ± 7.8 vs. 9.4 ± 4.8, *p* = 0.037). The presence of diabetes mellitus and paraprotein type (IgG vs. IgM) were not associated with differences in any autonomic domain (all *p* > 0.05). No differences were observed with respect to ongoing therapy. Higher autonomic symptom burden was associated with older age at the time of testing (*ρ* = 0.35, *p* = 0.012), greater muscle weakness as measured by the MRC‐SS (*ρ* = −0.58, *p* < 0.001), and increased functional disability, including higher INCAT lower limb scores (*ρ* = 0.47, *p* < 0.001), INCAT upper limb scores (*ρ* = 0.67, *p* < 0.001), and total INCAT scores (*ρ* = 0.65, *p* < 0.001). In a multivariable linear regression model adjusted for age, sex, and muscle strength, only overall disability (total INCAT score) remained independently associated with overall autonomic symptom burden (*β* = 0.42, *p* = 0.033) (Table S2B). This model explained approximately 45% of the variance in total SCOPA‐AUT scores (adjusted *R* ^2^ = 0.446, *p* < 0.001).

In CMT1A, no sex‐related differences in autonomic symptom burden in any of the domains were observed. Higher autonomic symptom burden was associated with greater muscle weakness as measured by the MRC‐SS (*ρ* = −0.34, *p* = 0.014) and increased functional disability, including higher ONLS upper limb scores (*ρ* = 0.57, *p* < 0.001), ONLS lower limb scores (*ρ* = 0.35, *p* < 0.001), and total ONLS scores (*ρ* = 0.53, *p* < 0.001), as well as greater overall disease severity as measured by the CMTES score (*ρ* = 0.43, *p* = 0.002). In a multivariable linear regression model adjusted for age, sex, and muscle strength, only overall functional disability (total ONLS score) remained independently associated with autonomic symptom burden (*β* = 0.68, *p* = 0.011; Table S2C). This model explained approximately 22% of the variance in total SCOPA‐AUT scores (adjusted *R* ^2^ = 0.222, *p* = 0.005).

## 4 Discussion

ANS involvement in chronic demyelinating polyneuropathies has not been systematically assessed to date [15]. In this large study comprising 218 patients with demyelinating polyneuropathies, we provide the first comprehensive characterization of ANS patterns across two common chronic immune‐mediated demyelinating polyneuropathies (CIDP and MGUS‐PNP) and two common hereditary demyelinating polyneuropathies (CMT1A and HNPP). We demonstrate that patients with CIDP, MGUS‐PNP, and CMT1A exhibit a substantial autonomic symptom burden, with distinct, disease‐specific ANS patterns.

Our patients with MGUS‐PNP had the most pronounced autonomic dysfunction when compared to HC. This is in contrast to the study of Klein et al., which found that autonomic testing was normal in 26 IgM‐MGUS patients [16]. This discrepancy might be due to different cohort characteristics, since we included only 2 MGUS‐PNP patients with IgM paraprotein in our study, while others had IgG or IgA monoclonal gammopathy. Beyond Klein et al., there is a paucity of studies quantifying autonomic symptom burden specifically in MGUS‐PNP, limiting direct comparability with our results. The autonomic patterns observed in our MGUS‐PNP patients were characterized by concurrent involvement of gastrointestinal, urinary, and thermoregulatory domains, suggesting a broader visceral autonomic pattern rather than selective system involvement. This differs from the more domain‐restricted autonomic changes observed in CIDP and supports the possibility that MGUS‐PNP and CIDP exhibit qualitatively distinct autonomic profiles. One potential interpretation is preferential involvement of small autonomic fibers across multiple visceral systems in MGUS‐PNP, either through immune‐mediated mechanisms or through a systemic plasma cell‐related process. MGUS‐PNP can also present as a sensory or sensorimotor axonal neuropathy [17], which may explain the involvement of thinly myelinated or unmyelinated autonomic nerve fibers. Notably, generalized autonomic dysfunction is a well‐established feature of light chain (AL) amyloidosis and represents an important clinical red flag when distinguishing amyloid neuropathy from other causes of polyneuropathy [18]. Accordingly, our findings raise the hypothesis that a subset of MGUS‐PNP patients may share elements of an amyloid‐spectrum autonomic pattern, albeit with milder or subclinical manifestations, warranting targeted evaluation in future studies. Interestingly, female patients with MGUS‐PNP in our study exhibited a lower overall autonomic symptom burden compared with male patients in the subgroup analysis. In this context, a large registry‐based analysis reported lower excess mortality in females with MGUS compared with males [19]. It may therefore be hypothesized that sex‐related differences in autonomic dysfunction could contribute, at least in part, to this survival advantage. We found that autonomic symptom burden relates to overall functional disability independently of demographic factors and motor weakness, suggesting that autonomic symptom burden represents a clinically relevant and distinct dimension of disease severity in MGUS‐PNP. These associations might, however, be bidirectional.

In CIDP, a recent literature review encompassing 12 studies and a total of 346 patients showed that clinical or subclinical ANS involvement is common but generally mild [15]. However, among these studies, only one assessed the severity of autonomic symptom burden using the Composite Autonomic Symptom Score‐31 (COMPASS‐31), and this study did not include a comparison with HC [20]. Notably, Pasangulapati et al. [20] reported COMPASS‐31 scores that were even lower than those observed in studies validating translated versions of the original COMPASS‐31 questionnaire in healthy populations [15, 21, 22]. These findings contrast with our results, which demonstrate a substantial overall autonomic symptom burden in CIDP patients compared with HC. In our cohort, CIDP patients predominantly exhibited more selective, thermoregulatory ANS involvement. This is consistent with the study by Figueroa et al., who reported mild autonomic dysfunction in 47 CIDP patients, characterized by cholinergic and predominantly sudomotor involvement [23]. Nevertheless, these observations require confirmation in larger, preferably multicenter cohorts. Rzepiński et al. reported in their review that autonomic dysfunction showed no correlation with disease severity, variant, or duration in CIDP patients [23]. Similarly, we found that clinical measures in CIDP (including muscle strength and functional disability) were not independent predictors of autonomic symptom burden, and they explained only a small percentage of autonomic score variability. This suggests that further investigations of other factors associated with autonomic dysfunction in CIDP should be performed.

We found that patients with CMT1A exhibited a higher autonomic symptom burden compared with HC, with thermoregulatory symptoms and sexual dysfunction in female patients showing the most pronounced relative involvement. Previous studies have suggested the presence of subclinical autonomic abnormalities in CMT1A [24, 25, 26]; however, comprehensive and systematic evaluation of ANS involvement remains limited. In contrast, no significant autonomic involvement was observed in patients with HNPP compared with HC. This finding was anticipated, as HNPP typically represents a milder phenotype affecting individual nerves within the spectrum of *PMP22*‐related peripheral neuropathies [27]. Similar to MGUS, autonomic symptom burden is related to overall disease severity in CMT1A, suggesting that autonomic symptom burden represents a clinically relevant and distinct dimension of disease severity in CMT1A that is not usually captured.

Our study has several limitations that should be acknowledged. The main limitation is the absence of objective ANS assessments. Autonomic symptom burden was evaluated using self‐reported questionnaires, which may not fully reflect objective autonomic dysfunction and may be influenced by recall or reporting bias. However, it is possible that autonomic impairments noticed by patients are even more relevant to patients and their quality of life than subclinical findings captured by instruments. The primary aim of this study was not to reassess the presence of autonomic involvement in chronic demyelinating polyneuropathies, but rather to characterize disease‐specific autonomic symptom patterns across these conditions. In addition, the cross‐sectional study design precludes conclusions regarding causality, and the relatively small sample sizes of certain subgroups, particularly HNPP, may have limited statistical power. Longitudinal studies incorporating objective autonomic testing in larger, multicentric cohorts are therefore warranted to validate these findings and to clarify their clinical relevance. Furthermore, assessing the impact of autonomic symptoms on quality of life may help to better define their clinical significance and relevance for patient management.

In conclusion, patients with CIDP, MGUS‐PNP, and CMT1A exhibit a substantial autonomic symptom burden with distinct, disease‐specific ANS patterns. Functional disability emerged as an independent predictor of greater autonomic symptom burden in MGUS‐PNP and CMT1A, underscoring the clinical relevance of autonomic involvement in these conditions and suggesting that autonomic impairment may be inherent to these diseases. Further studies incorporating longitudinal designs and objective autonomic assessments are warranted to confirm these findings and to clarify their clinical implications.

## Funding

The authors have nothing to report.

## Conflicts of Interest

The authors declare no conflicts of interest.

## Supporting information


**Table S1:** Post hoc pairwise comparisons for significant sociodemographic and clinical variables between five study groups.
**Table S2:** Multivariable linear regression analyses (enter method) of factors associated with overall autonomic symptom burden (total SCOPA‐AUT).

## Data Availability

Data are available upon reasonable request.
