# Supplementary material for: Autonomic Nervous System Phenotyping Across Chronic Demyelinating Peripheral Neuropathies: A Comparative Study
Source: J Peripher Nerv Syst. 2026 Jun 19;31(2):e70135. doi: 10.1111/jns.70135 (PMC13282177; doi:10.1111/jns.70135)
Supplement: Supplementary file 1 — Table S1: Post hoc pairwise comparisons for significant sociodemographic and clinical variables between five study groups. Table S2: Multivariable linear regression analyses (enter method) of factors associated with overall autonomic symptom burden (total SCOPA‐AUT). [file JNS-31-0-s001.docx]

Supplementary material

Supplementary table S1. Post hoc pairwise comparisons for significant sociodemographic and clinical variables between five study groups

| Features | Adjusted p-value |
| --- | --- |
| Sex | CIDP vs. MGUS: **0.440**  CIDP vs. CMT1A: **0.027**  CIDP vs. HNPP: **0.419**  CIDP vs. HC: **0.070**  MGUS vs. CMT1A: **0.010**  MGUS vs. HNPP: **0.210**  MGUS vs. HC: **0.024**  CMT1A vs. HNPP: **0.516**  CMT1A vs. HC: **0.410**  HNPP vs. HC: **0.874** |
| Age at the time of testing | CIDP vs. MGUS: **0.019**  CIDP vs. CMT1A: **0.005**  CIDP vs. HNPP: **<0.001**  CIDP vs. HC: **1.000**  MGUS vs. CMT1A: **<0.001**  MGUS vs. HNPP: **<0.001**  MGUS vs. HC: **0.011**  CMT1A vs. HNPP: **0.031**  CMT1A vs. HC: **0.004**  HNPP vs. HC: **<0.001** |
| Disease duration | CIDP vs. MGUS: **0.011**  CIDP vs. CMT1A: **<0.001**  CIDP vs. HNPP: **0.007**  MGUS vs. CMT1A: **<0.001**  MGUS vs. HNPP: **<0.001**  CMT1A vs. HNPP: **1.000** |
| MRC-SS | CIDP vs. MGUS: **1.000**  CIDP vs. CMT1A: **<0.001**  CIDP vs. HNPP: **1.000**  MGUS vs. CMT1A: **0.006**  MGUS vs. HNPP: **1.000**  CMT1A vs. HNPP: **0.009** |

Post hoc pairwise comparisons were performed using Dunn’s test with Bonferroni correction; CIDP – chronic inflammatory demyelinating polyneuropathy; MGUS – monoclonal gammopathy of undetermined significance–associated polyneuropathy; CMT1A – Charcot-Marie-Tooth type 1A; HNPP – hereditary neuropathy with liability to pressure palsies; HC – healthy controls; MRC-SS – Medical Research Council Sum Score

Supplementary table S2. Multivariable linear regression analyses (enter method) of factors associated with overall autonomic symptom burden (total SCOPA-AUT)

A. CIDP (n = 98)

| Predictor | B (SE) | Standardized β | p value | 95% CI for B |
| --- | --- | --- | --- | --- |
| Sex (female vs male) | 1.06 (1.94) | 0.054 | 0.586 | −2.79 to 4.91 |
| Age (years) | 0.08 (0.07) | 0.111 | 0.270 | −0.06 to 0.23 |
| MRC-SS | −0.33 (0.23) | −0.248 | 0.155 | −0.78 to 0.13 |
| Total INCAT score | 0.81 (0.79) | 0.178 | 0.306 | −0.75 to 2.37 |
| Model fit: R^2^ = 0.190; adjusted R^2^ = 0.155; F(4,93) = 5.46; p < 0.001 | | | | |

CIDP – Chronic inflammatory demyelinating polyneuropathy; MRC-SS – Medical Research Council sum score; INCAT – Inflammatory Neuropathy Cause and Treatment disability score; B – Unstandardized regression coefficient; SE – Standard error; β – Standardized regression coefficient; CI – Confidence interval; R^2^ – Coefficient of determination

B. MGUS-PNP (n = 51)

| Predictor | B (SE) | Standardized β | p value | 95% CI for B |
| --- | --- | --- | --- | --- |
| Sex (female vs male) | −3.06 (1.66) | −0.202 | 0.071 | −6.39 to 0.28 |
| Age (years) | 0.08 (0.08) | 0.127 | 0.293 | −0.07 to 0.24 |
| MRC-SS | −0.15 (0.14) | −0.191 | 0.295 | −0.43 to 0.14 |
| Total INCAT score | 1.69 (0.77) | 0.421 | **0.033** | 0.15 to 3.23 |
| Model fit: R^2^ = 0.491; adjusted R^2^ = 0.446; F(4,45) = 10.86; p < 0.001 | | | | |

MGUS-PNP – monoclonal gammopathy of undetermined significance–associated polyneuropathy; MRC-SS – Medical Research Council sum score; INCAT – Inflammatory Neuropathy Cause and Treatment disability score; B – Unstandardized regression coefficient; SE – Standard error; β – Standardized regression coefficient; CI – Confidence interval; R^2^ – Coefficient of determination

C. CMT1A (n = 51)

| Predictor | B (SE) | Standardized β | p value | 95% CI for B |
| --- | --- | --- | --- | --- |
| Sex (female vs male) | −1.15 (2.56) | −0.061 | 0.656 | −6.29 to 4.00 |
| Age (years) | −0.11 (0.12) | −0.156 | 0.354 | −0.35 to 0.13 |
| MRC-SS | 0.02 (0.34) | 0.013 | 0.954 | −0.66 to 0.70 |
| ONLS total score | 3.69 (1.39) | 0.678 | **0.011** | 0.90 to 6.48 |
| CMTES score | −0.07 (0.45) | −0.042 | 0.873 | −0.97 to 0.83 |
| Model fit: R^2^ = 0.300; adjusted R^2^ = 0.222; F(5,45) = 3.85; p = 0.005 | | | | |

CMT1A – Charcot-Marie-Tooth Examination Score; MRC-SS – Medical Research Council sum score; ONLS – Overall Neuropathy Limitations Scale; CMTES – Charcot-Marie-Tooth Examination Score; B – Unstandardized regression coefficient; SE – Standard error; β – Standardized regression coefficient; CI – Confidence interval; R^2^ – Coefficient of determination
